# Case Report: Crying-triggered pallid and hypotonic episodes in a 6-week-old infant with SARS-CoV-2 infection—a diagnostic dilemma

**DOI:** 10.3389/fped.2026.1989655

**Published:** 2026-10-22

**Authors:** Shun Wang, Sainan Chen, Yuqing Wang

**Affiliations:** Department of Respiratory Medicine, Children's Hospital of Soochow University, Suzhou, Jiangsu, China

**Keywords:** apnea, breath-holding spells, case report, infant, SARS-CoV-2

## Abstract

Crying-triggered pallid and hypotonic episodes in early infancy can be difficult to classify. We report a 44-day-old term female infant with acute severe acute respiratory syndrome coronavirus 2 (SARS-CoV-2) infection who experienced two crying-triggered episodes of marked pallor, limpness, and reduced responsiveness after cessation of crying, followed by spontaneous recovery. Neither event was physiologically recorded, and a definitive cause could not be established. No further episodes were reported during hospitalization or 8 weeks of follow-up. The clinical sequence suggested breath-holding spells (BHS) as one possible explanation alongside infection-associated apnea and other causes of paroxysmal events in early infancy. This case highlights the need for careful differential diagnosis of crying-triggered pallid and hypotonic episodes occurring during acute SARS-CoV-2 infection.

## 1 Introduction

Paroxysmal episodes of pallor and hypotonia in young infants have a broad differential diagnosis. Breath-holding spells (BHS) are paroxysmal nonepileptic events that predominantly affect healthy infants and young children. Cyanotic and pallid subtypes are recognized, typically triggered by emotional distress or crying and by pain or fear, respectively (1–3). BHS most commonly begin between 6 and 18 months of age, and presentation before 6 months is uncommon; however, cases occurring during the neonatal period and early infancy have occasionally been reported, including onset within the first days of life (1, 2, 4, 5). Apnea and cyanosis have also been reported in young infants with severe acute respiratory syndrome coronavirus 2 (SARS-CoV-2) infection (6–8). In this age group, however, crying-triggered paroxysmal events may have overlapping clinical features and can be difficult to classify. We report a 44-day-old infant who experienced two crying-triggered pallid and hypotonic episodes during acute SARS-CoV-2 infection. We describe the evaluation of these events and the features that prevented a definite diagnosis, with BHS considered as one possible explanation. This case highlights the need to consider the possibility of unusually early BHS when evaluating crying-triggered paroxysmal events in young infants with SARS-CoV-2 infection.

## 2 Case presentation

A 44-day-old female infant was admitted because of a 1-day history of mild cough, nasal congestion; the highest rectal temperature prior to admission was 37.5 °C. The cough consisted of one or two coughs at a time, occurring approximately every 3–4 h, without a characteristic inspiratory whoop. She had been born at term by vaginal delivery with a birth weight of 3,050 g and an Apgar score of 10 at both one and five minutes. There was no history of perinatal asphyxia or birth injury. She had been breastfed since birth, with appropriate growth and development and no history of recurrent choking, regurgitation, or feeding-related events. There was no family history of similar spells, epilepsy, arrhythmia, long-QT syndrome, or sudden unexplained death, and no history of consanguinity. Both parents had preceding respiratory symptoms, including fever, cough, and nasal congestion, but were not tested for SARS-CoV-2; there was no known household history of pertussis. Her 20-month-old brother was healthy.

The clinical course is summarized in Figure 1. During this illness, the infant experienced two paroxysmal events associated with crying rather than feeding or coughing. The first event occurred in the morning. Crying stopped and was followed by marked pallor of face and lips, generalized limpness, staring and complete unresponsiveness. No rhythmic limb movements, tonic stiffening, jaw clenching, oral frothing, or urinary or fecal incontinence were observed. Breath-holding or apnea was not specifically observed. No preceding cyanosis was reported. The infant remained unresponsive despite caregiver patting. The staring and unresponsiveness resolved after approximately 2 min, while pallor persisted briefly. The duration was estimated retrospectively by the caregiver rather than objectively timed. After the first event, the infant was taken to another hospital and admitted. A second similar event, also approximately 2 min, occurred there that afternoon, approximately 4 h after the first. The day after the second event, the family brought her to our hospital for further evaluation. Neither event was associated with feeding, choking, regurgitation or vomiting.

**Figure 1:**
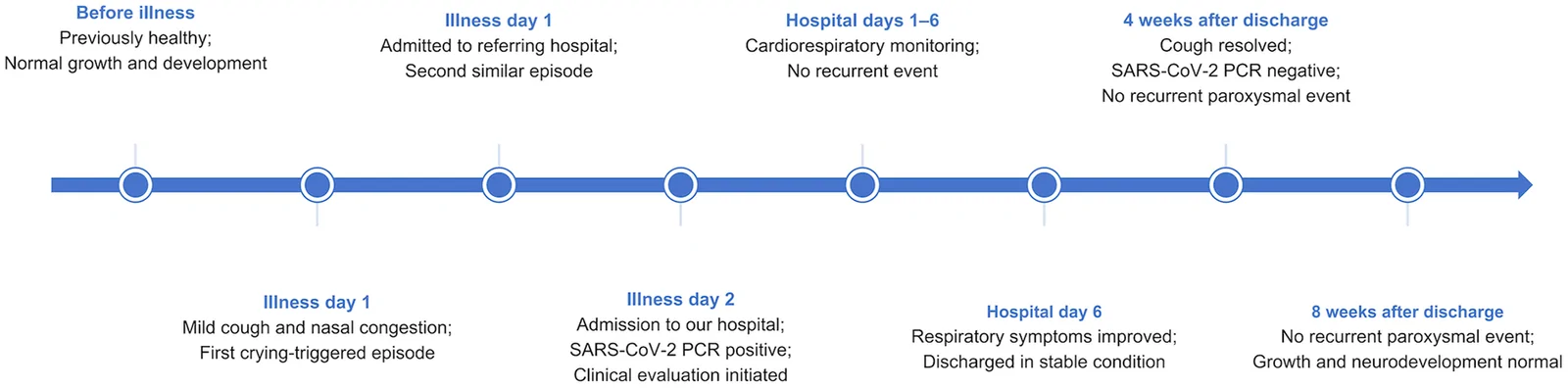
Clinical timeline from onset of respiratory symptoms and paroxysmal events through hospitalization and follow-up.

Initial laboratory testing showed a white blood cell count of 7.5 × 10^9^/L, with lymphocytes accounting for 63.6% (absolute lymphocyte count, approximately 4.77 × 10^9^/L), hemoglobin of 126 g/L, and high-sensitivity C-reactive protein <0.2 mg/L. Urinalysis was unremarkable, with negative leukocyte esterase and nitrite, no pyuria or bacteriuria, and negative for both white blood cells and red blood cells. Urine and blood cultures, procalcitonin testing, and lumbar puncture were not performed. Blood glucose, blood gas, lactate, ammonia, and serum electrolytes showed no clinically relevant abnormalities. Cranial ultrasonography was normal. Electrocardiography (ECG) obtained while the infant was crying showed sinus tachycardia at approximately 200 beats/minute, with manually measured PR interval of 110 ms, QRS duration of 70 ms, QT interval of 180 ms, and a Bazett-corrected QT interval (QTc) of 330 ms. The tracing did not capture a paroxysmal event. Echocardiography demonstrated a patent foramen ovale and mild tricuspid regurgitation, without significant structural or functional cardiac abnormality.

At admission, the infant was afebrile and clinically stable. Heart rate was 136 beats/min, respiratory rate 34 breaths/min, blood pressure 78/57 mmHg, and SpO_2_ 99% on room air. She was alert and responsive, with normal respiratory effort, muscle tone, and spontaneous movements. The anterior fontanelle was soft and flat, and there was no persistent irritability, lethargy, vomiting, or focal neurological abnormality. There was no reported history of trauma. Skin and mucosal examination showed no bruising or subcutaneous bleeding. The pupils were equal and reactive, with preserved reflexes, and no focal neurological or meningeal signs.

SARS-CoV-2 RNA was detected by fluorescence PCR from a nasopharyngeal swab. A nasopharyngeal aspirate was obtained using a sterile catheter inserted through the nasal cavity to the lower pharynx, and used for a 13-pathogen respiratory panel and bacterial culture (9). Both tests were negative.

Pediatric neurological consultation considered the episodes clinically suggestive of BHS, although the unusually early age and atypical presentation prompted further neurological evaluation. Brain magnetic resonance imaging (MRI) was recommended to exclude structural intracranial abnormalities but was declined by the caregivers because of concerns about sedation. Video electroencephalography (video-EEG) was planned if another episode occurred or if abnormal movements developed. Neither recurrent events nor abnormal motor activity were observed during hospitalization, and video-EEG was therefore not performed.

The infant remained clinically stable under cardiorespiratory monitoring, with no recurrent pallid or cyanotic event, clinically apparent apnea, bradycardia, or oxygen desaturation. Supportive treatment was provided for the respiratory infection, and neither antiseizure medication nor apnea-specific treatment was used. The diagnostic evaluation and key findings are summarized in Table 1.

**Table 1:** Summary of diagnostic evaluation.

**System evaluated**	**Investigation**	**Main finding**
Infectious	SARS-CoV-2 PCR	Positive
Infectious	13-pathogen respiratory panel and bacterial culture of nasopharyngeal aspirate	Negative
Infectious	Bordetella pertussis PCR	Not performed
Infectious	Urinalysis	No evidence of urinary tract inflammation
Infectious	Urine and blood cultures	Not performed
Infectious	Lumbar puncture	Not performed
Hematologic	CBC, hs-CRP	WBC 7.5 × 10^9^/L, lymphocytes 63.6%, hs-CRP <0.2 mg/L
Hematologic	Hemoglobin	126 g/L
Hematologic	Ferritin and other iron indices	Not performed
Metabolic	Glucose, blood gas, lactate, ammonia, electrolytes	No clinically relevant abnormality
Neurological	Neurological examination	Normal between episodes
Neurological	Cranial ultrasonography	No significant abnormality
Neurological	Brain MRI	Recommended because of atypical early presentation; declined by caregivers
Neurological	Video-EEG	Planned if recurrence or abnormal movements developed; not performed
Cardiac	ECG obtained during crying	Sinus tachycardia; QTc 330 ms
Cardiac	Echocardiography	Patent foramen ovale and mild tricuspid regurgitation; no other significant abnormality reported
Cardiac	Holter monitoring	Not performed
Inpatient monitoring	Bedside cardiorespiratory monitoring	No documented recurrent event, bradycardia, or desaturation

SARS-CoV-2, severe acute respiratory syndrome coronavirus 2; The 13-pathogen respiratory panel included influenza A virus H1N1, influenza A virus, seasonal H3N2 virus, influenza B virus, parainfluenza virus, respiratory syncytial virus, human rhinovirus, human coronavirus, human metapneumovirus, Chlamydia pneumoniae, Mycoplasma pneumoniae, adenovirus, and human bocavirus; CBC, complete blood count; hs-CRP, high-sensitivity C-reactive protein; WBC, white blood cell; MRI, magnetic resonance imaging; Video-EEG, video electroencephalography; ECG, electrocardiography.

The respiratory symptoms gradually improved, and the infant was discharged in good general condition after 6 days of hospitalization. At the 4-week outpatient follow-up, the cough had completely resolved and repeat SARS-CoV-2 PCR testing was negative. At 8-week follow-up, no further crying-triggered pallid or hypotonic episodes, altered responsiveness, or suspected apnea had been reported. Growth and neurodevelopment remained age appropriate.

## 3 Discussion

This case illustrates the difficulty of classifying crying-triggered paroxysmal events during early infancy. The episodes share features considered in the brief resolved unexplained event (BRUE) framework, including pallor, hypotonia, and reduced responsiveness (10, 11). The infant had several higher-risk features within the BRUE framework, including age <60 days, two reported episodes, and a caregiver-estimated duration exceeding 1 min. These features meant that she did not meet the lower-risk criteria. Furthermore, the reported duration and concurrent respiratory illness complicated formal classification as BRUE. These features supported inpatient observation and broader neurological, cardiac, respiratory, and infectious evaluation.

BHS is primarily diagnosed clinically based on a characteristic sequence of crying or another precipitating event, color change, altered tone or responsiveness, and spontaneous recovery (2, 3). Although breath-holding itself was not directly observed, the crying-related onset, marked pallor, limpness, reduced responsiveness, and subsequent recovery had features that could suggest BHS. However, several features were atypical. The phenotype also did not fit either the classical cyanotic or pallid subtypes. Crying is more commonly associated with cyanotic BHS, whereas the prominent pallor in this infant resembled a pallid phenotype, which is more typically precipitated by pain or fear. The events began at approximately 6 weeks of age, and the caregiver-reported duration of about 2 min exceeded the usual 60 s reported for classical BHS (2, 3). Overall, the clinical sequence was suggestive of BHS, although the unusually young age, caregiver-estimated duration, absence of directly observed breath-holding, and lack of event-specific physiological recording prevented definitive classification as BHS.

Very early BHS has nevertheless been described. Breukels et al. reported onset at 3 days of age in an infant with a positive family history, while Bhat and Ali described neonatal cyanotic BHS without familial predisposition (4, 5). In the recent case reported by Alosail et al., expiratory breath-holding and oxygen desaturation were objectively documented, and persistence of the episodes with later evolution into a classical emotion-triggered pattern supported the diagnosis of neonatal-onset BHS (1). In contrast, our patient had no event-specific physiological recording and no recurrence during hospitalization or short-term follow-up. The absence of recurrence does not exclude BHS, but it provides no longitudinal confirmation and leaves open the possibility that the episodes represented an infection-related or other transient phenomenon. Respiratory inhibition after crying (RIAC) is another consideration in early infancy because it is characterized by respiratory inhibition and hypoxemia following crying (1, 12). No hypoxemia was documented during the events in our patient, and no similar episodes were reported after recovery, although the short duration of follow-up limits firm distinction between RIAC and early BHS. Iron deficiency is another recognized association with BHS (2, 13). Although the infant was not anemic, ferritin and other iron indices were not measured, so iron deficiency without anemia could not be excluded.

SARS-CoV-2-associated apnea is an important alternative diagnosis in this age group. Loron et al. described recurrent apneas with absent respiratory movements, oxygen desaturation to 70%, and a need for tactile stimulation (14). Similar presentations have also been reported in neonates and young infants with COVID-19 (6, 15). In our patient, cessation of breathing was not witnessed and no physiological data were recorded during either event. Instead, the caregivers described a similar sequence of crying, pallor, limpness, and reduced responsiveness. The clinical sequence raised BHS as one possible explanation alongside infection-associated apnea, although brief apnea during the episodes cannot be excluded.

The relationship between SARS-CoV-2 infection and the episodes remains uncertain. Both events occurred during the acute illness, and SARS-CoV-2 fluorescence PCR was positive. The absence of recurrence after recovery may be compatible with a transient illness-related phenomenon, but the available evidence did not establish whether the infection contributed to them. Very early BHS can also occur in the absence of infection (1, 4, 5). The available evidence therefore cannot determine whether SARS-CoV-2 contributed to the episodes or was coincidental.

Pertussis is another important infectious consideration in a young infant presenting with cough and possible apnea or color change. Although infants with pertussis may lack the characteristic inspiratory whoop, the illness typically progresses to a prolonged period of paroxysmal coughing (16). In our patient, the cough was mild and intermittent, both paroxysmal events followed crying rather than coughing, and the cough had resolved by the 4-week follow-up. The white blood cell count was 7.5 × 10^9^/L, with an absolute lymphocyte count of approximately 4.77 × 10^9^/L, without the marked leukocytosis or absolute lymphocytosis often seen in infant pertussis. These findings made typical pertussis less likely. However, specific Bordetella pertussis PCR was not performed, and an atypical or early presentation could not be definitively excluded.

The 2021 AAP guideline for febrile infants aged 8–60 days applies to infants with a documented rectal temperature of ≥38.0 °C (17). In our patient, the highest reported temperature was 37.5 °C, and she remained clinically well appearing. Urinalysis was negative for leukocyte esterase, nitrite, pyuria, and bacteriuria, making urinary tract infection unlikely. The normal white blood cell count, low high-sensitivity C-reactive protein, and reassuring clinical course also provided little evidence of an invasive bacterial infection. Nevertheless, blood and urine cultures, procalcitonin testing, and lumbar puncture were not performed, so bacterial infection was not completely excluded.

Epileptic seizure was considered less likely because the episodes followed crying and were characterized by pallor and limpness with spontaneous recovery, without tonic-clonic movements or a clear postictal state (2, 3). Hyperekplexia was also considered, but neonatal hypertonia, exaggerated startle, and stimulus-induced generalized stiffness were absent (18). Abusive head trauma was also considered given the infant's young age and unexplained paroxysmal events. There was no reported trauma history, and skin and neurological examinations revealed no findings suggestive of injury. Cranial ultrasonography was unremarkable. Thus, the available clinical findings did not raise specific suspicion of intracranial injury. Nevertheless, occult abusive head trauma could not be fully excluded because cranial ultrasonography has limited sensitivity and MRI, fundoscopy, and skeletal survey were not performed. Gastroesophageal reflux was considered less likely because there was no history of recurrent regurgitation, choking, or poor weight gain. And the reported events were not associated with feeding, choking, or regurgitation. Cardiac causes, including intermittent arrhythmia and long-QT syndrome (19), were also considered. The available ECG was recorded during crying and showed sinus tachycardia. Echocardiography revealed only a patent foramen ovale and mild tricuspid regurgitation, without significant structural or functional abnormality. No recurrent event, bradycardia, or clinically apparent arrhythmia was documented during 6 days of inpatient cardiorespiratory monitoring. These findings provided no evidence of a significant structural cardiac disorder or persistent arrhythmia. However, neither presenting event was captured on ECG, and Holter monitoring was not performed. An intermittent arrhythmic cause therefore could not be definitely excluded.

## 4 Limitations

Several limitations should be considered. Neither episode was witnessed by the treating team or captured on video, and the reported duration relied on caregiver recall. Recordings of breathing, oxygen saturation, and heart rhythm were not available during either event. Ferritin and other iron indices were not measured, so iron deficiency without anemia could not be assessed. Specific testing for pertussis was not performed; therefore, pertussis could not be definitively excluded as an alternative infectious explanation. Brain MRI and video-EEG were not obtained, and dedicated evaluation for occult abusive head trauma was not performed. Follow-up was limited to 8 weeks, which is insufficient to determine whether a recurrent or more typical pattern would develop. Longer longitudinal observation is therefore needed.

## 5 Key learning points

- Crying-triggered pallid and hypotonic episodes in very young infants require a broad differential diagnosis, including BHS, apnea, neurological events, cardiac causes, and infectious conditions.
- BHS usually begin after 6 months of age, although neonatal and early-infant presentations have been reported. However, atypical features such as very young age, pallor after crying, absence of directly observed breath-holding, and prolonged caregiver-estimated duration should limit diagnostic certainty.
- Apnea can occur in young infants with SARS-CoV-2 infection, but cessation of crying alone does not establish apnea.
- Careful event characterization, age-appropriate evaluation, and longitudinal follow-up are important when the initial diagnosis remains uncertain.

## 6 Conclusion

This case illustrates the diagnostic uncertainty surrounding crying-triggered pallid and hypotonic episodes in a 6-week-old infant during acute SARS-CoV-2 infection. Although the clinical sequence showed several features compatible with BHS, the atypical presentation and absence of recordings of breathing, oxygen saturation, or heart rhythm during an event precluded definitive classification. The temporal association of infection and the episodes does not establish a causal relationship, and BHS should remain a consideration within a broader differential diagnosis that includes infection-associated apnea and other neurological, cardiac, and infectious causes.

## Patient perspective

We were very frightened when she suddenly became pale and limp after crying and did not know whether she was having a serious problem. The doctors monitored her closely during her hospital stay, and we were relieved that the episodes did not recur. We have continued to watch her carefully at home and have not seen another similar episode.

## Data Availability

The original contributions presented in the study are included in the article/Supplementary Material, further inquiries can be directed to the corresponding author.

## References

[1] Alosail A Alotaibi A Jarwann R AlHojailan A AlRashed M AlFadhel A. Neonatal-onset cyanotic breath-holding spells: a case report with longitudinal diagnostic dilemma. Front Pediatr. (2026) 14:1783586. 10.3389/fped.2026.1783586 42023281 PMC13095757

[2] Leung AKC Leung AAM Wong AHC Hon KL. Breath-holding spells in pediatrics: a narrative review of the current evidence. Curr Pediatr Rev. (2019) 15(1):22–9. 10.2174/1573396314666181113094047 30421679 PMC6696822

[3] Hellström Schmidt S Smedenmark J Jeremiasen I Sigurdsson B Eklund EA Pronk CJ. Overuse of EEG and ECG in children with breath-holding spells and its implication for the management of the spells. Acta Paediatr. (2024) 113(2):317–26. 10.1111/apa.17020 37905418

[4] Bhat MA Ali W. Cyanotic breath holding spell in a neonate. A rare entity. Neurosciences (Riyadh). (2008) 13(2):190–1. 10.17712/1658-3183.1591 21063322

[5] Breukels MA Plötz FB Van Nieuwenhuizen O Van Diemen-Steenvoorde JA. Breath holding spells in a 3-day-old neonate: an unusual early presentation in a family with a history of breath holding spells. Neuropediatrics. (2002) 33(1):41–2. 10.1055/s-2002-23591 11930276

[6] Paolin C Zanetto L Frison S Boscolo Mela F Tessari A Amigoni A. Apneas requiring respiratory support in young infants with COVID-19: a case series and literature review. Eur J Pediatr. (2023) 182(5):2089–94. 10.1007/s00431-023-04856-x 36912961 PMC10009862

[7] Krajcar N Marić LS Roglić S Tešović G. Among young infants with uncomplicated COVID-19: should we broaden diagnostic tests for infectious causes of apnea? Pediatr Infect Dis J. (2022) 41(7):e301–e2. 10.1097/INF.0000000000003536 35389947 PMC9177123

[8] Needleman JS Hanson AE. COVID-19-associated apnea and circumoral cyanosis in a 3-week-old. BMC Pediatr. (2020) 20(1):382. 10.1186/s12887-020-02282-8 32787956 PMC7419228

[9] Gu W Li Z Han E Kuai X Hua S Gao S. Genotyping and refractory risk factors of mycoplasma pneumoniae pneumonia in suzhou, China. BMC Infect Dis. (2025) 25(1):561. 10.1186/s12879-025-10964-w 40251512 PMC12008929

[10] Tieder JS Bonkowsky JL Etzel RA Franklin WH Gremse DA Herman B. Brief resolved unexplained events (formerly apparent life-threatening events) and evaluation of lower-risk infants. Pediatrics. (2016) 137(5):e20160590. 10.1542/peds.2016-0590 27244835

[11] Merritt JL 2nd Quinonez RA Bonkowsky JL Franklin WH Gremse DA Herman BE. A framework for evaluation of the higher-risk infant after a brief resolved unexplained event. Pediatrics. (2019) 144(2):e20184101. 10.1542/peds.2018-4101 31350360

[12] Minowa H. Neonatal respiratory inhibition. J Matern Fetal Neonatal Med. (2022) 35(25):7132–8. 10.1080/14767058.2021.1944094 34182876

[13] Aslan G Alkan F Bircan O Coskun S. Effects of iron replacement on electrocardiogram parameters in breath-holding spells patients with iron deficiency. BMC Pediatr. (2025) 25(1):865. 10.1186/s12887-025-06204-4 41146111 PMC12557941

[14] Loron G Tromeur T Venot P Beck J Andreoletti L Mauran P. COVID-19 associated with life-threatening apnea in an infant born preterm: a case report. Front Pediatr. (2020) 8:568. 10.3389/fped.2020.00568 33042914 PMC7522361

[15] Tamayo-Múnera C Insignares Vizcaino ZM Torres Millán AL Niño-Serna L Giraldo Alzate C Vanegas Diaz CA. Apnea or cyanosis as COVID-19 initial presentation in newborns. J Infect Dev Ctries. (2023) 17(10):1401–6. 10.3855/jidc.17846 37956375

[16] Pediatric Infection Group, Society of Infectious Diseases, Chinese Medical Association. [Guidelines for diagnosis and management and prevention of pertussis of China (2024 edition)]. Zhonghua yi xue za zhi. (2024) 104(15):1258–79. 10.3760/cma.j.cn112137-20240124-00179 38637166

[17] Pantell RH Roberts KB Adams WG Dreyer BP Kuppermann N O'Leary ST. Evaluation and management of well-appearing febrile infants 8 to 60 days old. Pediatrics. (2021) 148(2):e2021052228. 10.1542/peds.2021-052228 34281996

[18] Falsaperla R Sortino V Giacchi V Saporito MAN Marino S Tardino LG. Neonatal hyperekplexia: is it still a diagnostic challenge? Evidence from a systematic review. J Child Neurol. (2024) 39(11-12):415–24. 10.1177/08830738241273425 39223854

[19] Zeppenfeld K Tfelt-Hansen J De Riva M Winkel BG Behr ER Blom NA. 2022 ESC guidelines for the management of patients with ventricular arrhythmias and the prevention of sudden cardiac death. Eur Heart J. (2022) 43(40):3997–4126. 10.1093/eurheartj/ehac262 36017572

